# A(H1N1) pandemic influenza and its prevention by vaccination: Paediatricians' opinions before and after the beginning of the vaccination campaign

**DOI:** 10.1186/1471-2458-11-128

**Published:** 2011-02-22

**Authors:** Eve Dubé, Defay Fannie, Gilca Vladimir, Bettinger A Julie, Sauvageau Chantal, Lavoie France, Boucher D François, McNeil Shelly, Gemmill Ian, Boulianne Nicole

**Affiliations:** 1 Institut National de Santé publique du Québec, 2400 d'Estimauville, Québec, Canada; 2 Centre de recherche du CHUL-CHUQ, Centre Hospitalier de l'Université Laval, 2705 Blv Laurier, Québec, Canada; 3 University of British Columbia, 229 West mall, Vancouver, British Columbia, Canada; 4 Canadian Center for Vaccinology, Health Centre, Clinical Trials Research Center, 5850/5980 University avenue, Halifax, Nova Scotia, Canada; 5 Kingston, Frontenac and Lennox & Addington Public health Unit, 221 Portsmouth avenue, Kingston, Ontario, Canada

## Abstract

**Background:**

In June 2009, the World Health Organization declared an A(H1N1) influenza pandemic. In October 2009, the largest vaccination campaign in Canadian history began. The aim of this study was to document paediatricians' knowledge, attitudes and practices (KAP) regarding A(H1N1) pandemic influenza and its prevention by vaccination just after the beginning of the A(H1N1) vaccination campaign and to compare the results with those obtained before campaign initiation.

**Methods:**

A self-administered mail-based questionnaire was sent to all Canadian paediatricians. Questionnaires were analyzed in two subsets: those received before and after the beginning of the vaccination campaign.

**Results:**

Overall the response rate was 50%. Respondents' characteristics were comparable between the two subsets. Before the beginning of the campaign, 63% of paediatricians perceived A(H1N1) pandemic infection as a serious disease, that would occur frequently without vaccination compared to more than 75% after. Before the vaccination campaign, half of respondents or less thought that the A(H1N1) vaccine was safe (50%) and effective (35%) compared to 77% and 72% after. The proportion of paediatricians who reported they had received sufficient information on A(H1N1) vaccine increased from 31% before to 73% after the beginning of the vaccination campaign. The majority of respondents intended to get vaccinated against A(H1N1) influenza themselves (84% before and 92% after). Respondents' intention to recommend the A(H1N1) vaccine to their patients increased from 80% before the beginning of the campaign to 92% after. In multivariate analysis, the main determinants of paediatricians' intention to recommend the A(H1N1) vaccine were their intention to get vaccinated against A(H1N1) influenza themselves and a belief that A(H1N1) vaccine would be well accepted by health professionals who administer vaccines to the public.

**Conclusion:**

Results of this study show important increases in physicians' level of confidence about A(H1N1) vaccine's safety and immunogenicity and their willingness to recommend this vaccine to their patients. These changes could be explained, at least partially, by the important effort done by public health authorities to disseminate information regarding A(H1N1) vaccination.

## Background

In June 2009, the World Health Organization (WHO) declared an A(H1N1) influenza pandemic [1]. In October 2009, the largest vaccination campaign in Canadian history began. Before the start of the A(H1N1) pandemic influenza vaccination campaign, limited information on the safety and immunogenicity of A(H1N1) influenza vaccines was available. However, the spread of A(H1N1) influenza generated intense media interest in pandemic preparedness and contradictory information around the vaccine and the vaccination campaign was reported [2-6]. During the first wave of A(H1N1) influenza in Canada, 77 deaths were reported, mostly in the provinces of Quebec and Ontario. During the second wave, in early fall 2009, 351 deaths were reported across the country [7]. In Canada, almost exclusively, an A(H1N1) pandemic influenza vaccine (Arepanrix™) containing a novel adjuvant (AS03 adjuvant, as an oil-in-water emulsion) was used [8].

Physicians are known to play a key role in public acceptance of new vaccines and their recommendations are an important determinant of vaccine uptake [9-13]. Prior to the A(H1N1) vaccine approval for clinical use and the release of professional association and experts committee recommendations, we documented Canadian family physicians' and paediatricians' knowledge, attitudes and practice (KAP) regarding A(H1N1) pandemic influenza and its prevention by vaccination [14]. In this study, 59% of paediatricians had had some experience with severe cases of A(H1N1) pandemic influenza and the majority (75%) of them were willing to recommend the A(H1N1) pandemic vaccine to their patients. More than 75% of the respondents also indicated the willingness to get the vaccine themselves [14].

The aim of this study was to document paediatricians' KAP regarding A(H1N1) pandemic influenza and its prevention by vaccination just after the beginning of the A(H1N1) vaccination campaign and to compare these results with those obtained before vaccination campaign initiation.

## Methods

A self-administered, anonymous, mail-based questionnaire was sent to all Canadian paediatricians, except subspecialist. The Canadian Medical Directory [15] was used to identify paediatricians. This database contains more than 58,000 listings of Canadian physicians medical contact information and is updated each year. A multidisciplinary team developed the questionnaire using the Analytical framework for immunization programs in Canada as a theoretical base [16]. This framework was developed to guide and standardize public health decision-making process regarding new immunization programs in Canada. It includes 58 criteria classified into 13 categories. Three categories of this framework were used to guide the construction of the questionnaire: (1) Burden of disease, (2) Vaccine characteristics, and (3) Acceptability of the vaccine program. The final questionnaire included 12 questions on A(H1N1) pandemic influenza and its prevention by vaccination as well as 10 questions on KAP about vaccination in general and 10 questions on demographic and professional characteristics of respondents. Respondents were asked to base their answers on their own knowledge and opinions. For most questions, a 6-point Likert answer scale ranging from "strongly disagree" to "strongly agree" was used. No information on A(H1N1) pandemic influenza or the vaccines was provided. The questionnaire was mailed to 1,852 pediatricians. The first two mailings were done in August-September 2009 and the third in November 2009. The last mailing was sent to 1,118 pediatricians who had not responded to the first two mailings. The study protocol was approved by the Ethics Board of the Laval University Hospital Center (reference number 126.05.02).

All vaccines authorized for sale in Canada, including the A(H1N1) influenza vaccines, are reviewed and approved by the federal government (Health Canada). However, each province and territory is responsible for the development of publicly funded immunization programs, including the schedules and the logistics of administering vaccines as well as education of the population and health professionals. The A(H1N1) pandemic vaccine was approved for use in Canada on 21^st ^October 2009 and vaccination campaign started shortly afterwards in all Canadian provinces and territories (within days before or after 29^th ^October 2009). To vaccinate as many persons as possible in the shortest period of time, most Canadian jurisdictions used mass vaccination centres administered by the public system. All provincial and territorial authorities, conjointly with federal authorities, have determined a sequence of vaccination by target groups. Throughout Canada, priority to receive the A(H1N1) vaccine was given to healthcare workers. Mass vaccination campaign ended in most Canadian provinces and territories in mid-December 2009.

Due to the intense media coverage and the important educational efforts undertaken around the vaccination campaign and their potential impact on physicians' KAP [17,18], we decided to perform a "before-after" analysis. Questionnaires were analyzed in two subsets: those received before (first subset) and after (second subset) the start of the vaccination campaign (October 29). Descriptive statistics were generated for all variables. Missing responses were excluded from the analyses. Univariate analyses were computed separately for the two data subsets. Comparisons of categorical responses were performed using chi-square or Fisher's exact tests. A multivariate logistic regression model was used to determine variables independently associated with the paediatrician's intention to recommend the A(H1N1) pandemic vaccine. Dependent and explanatory variables were dichotomized: the responses "strongly agree" and "agree" versus all others ("strongly disagree", "disagree", "somewhat disagree" and "somewhat agree"). Variables associated in the univariate analysis with the intention to recommend the vaccine at p ≤ 0.20 were entered into the multivariate regression models using the stepwise selection technique. The model was adjusted to take into consideration the two subsets of data. A new binary explanatory variable was created (subsequently referred to as "subset variable") and forced into the model: questionnaires mailed before the beginning of the vaccination campaign and questionnaires mailed after. Variables were reevaluated in the final model to check for confounding and model fit. A probability level of p < 0.05 based on two-sided tests was considered statistically significant. The collinearity was checked and the adequacy of the model was evaluated by Hosmer and Lemeshow's goodness of fit test.

Multiple correspondence analysis (MCA) method [19] was also used as a complementary way to analyse our dataset. MCA is used to detect links between variables (including, in this study, the intention to recommend the A(H1N1) vaccine), but there is no dependent variable. This method is a form of principal components analysis that is appropriate for qualitative variables. MCA searches for principal components, which are new quantitative modelled variables, constructed as linear functions of the initial variables. Finding the principal components is based on the maximisation of the correlation ratio between the principal component and the initial variables. All principal components are mutually uncorrelated by construction. Our initial variables were all variables in the questionnaire pertaining to A(H1N1) vaccine and A(H1N1) influenza and the subset variable. Analysis was computed using the raw variables (6 degrees of answers ranging from "Strongly agree" to "Strongly disagree"). We carried out this MCA as a sensitivity analysis to better assess the role of the subset variable and the impact of all 6 degrees of possible answers on the Likert scale. The Statistical Analysis Systems (SAS^®^) software (version 9.2 of the SAS system for Windows. Copyright (c) 2002-2008 by SAS Institute Inc., Cary, NC, USA) and R software (version 2.11.1) [20] with the library FactoMineR [21] were used for data analyses.

## Results

### Participation and socio-professionals characteristics

Overall, 912 paediatricians have completed the questionnaires: 714 completed the questionnaire before the beginning of the A(H1N1) vaccination campaign and 197 completed it after. After exclusion of physicians no longer practicing, those with incorrect addresses or those who were subspecialists, the overall participation rate was 50% (911/1832). Participation rates by country regions varied from 40.1% in Prairies to 57.7% in Quebec. Table 1 shows respondents' socio-professionals characteristics. No statistically significant differences were found among the characteristics of paediatricians who complete the survey before and after the beginning of A(H1N1) vaccination campaign.

**Table 1:** Paediatricians' professional and demographic characteristics (%)

Characteristics	*Before N = 714*	*After N = 197*	P-value
**Sex**			
Male	41.9	46.7	0.2327
**Location of main practice**			
Private office	44.6	42.3	
Hospital	51.8	52.1	0.4371
Other	3.7	5.7	
**Number of hours in outpatient consultation**			
<7 hours	16.7	24.9	'>21 hrs' vs. others:
From 7 to 21 hours	28.8	26.5	
>21 hours	54.5	48.6	0.1571
**Number of doses of vaccines administered each month in respondents' main practice place**			
None	42.1	48.9	
<30 doses	29.5	30.9	None vs. others: 0.0924
From 30 to 100 doses	13.1	6.4	
>100 doses	15.3	13.8	
**Number of years of practice**			
>20 years	39.6	37.9	
From 10 to 20 years	37.2	40.5	0.6915
<10 years	23.2	21.6	
**Provinces of practice**			
Atlantic	9	7.2	
Quebec	29.6	22.2	
Ontario	35.1	40.7	0.2067
Prairies	16.2	17.5	
British Columbia	10.1	12.4	
**I am planning to get vaccinated against A(H1N1) pandemic influenza**			
Yes	83.9	92.3	0.0028
No - don't know	16.2	7.7	

### Knowledge, attitudes and practices regarding vaccination in general

Overall, 98% of paediatricians thought that vaccines recommended by public health authorities are very useful (97,6% before and 98,5% after; p = 0,5911), and 73% agreed or strongly agreed with the statement "it is very useful to protect children with the vaccines against seasonal influenza" (72,9% before and 73,9% after; p = 0,7855). When recommending new vaccines to their patients, 91% of paediatricians indicated that they are highly influenced by expert group recommendations (91,3% before and 90,7% after; p = 0,8152) and 90%, by professional association recommendations (90,8% before and 89,7% after; p = 0,6414). Approximately half of paediatricians (49%) stated that it is easy for them to advise their patients on new vaccines. No statistically significant differences were found in attitudes towards vaccination in general between respondents who answered before or after the beginning of A(H1N1) vaccination campaign

### Knowledge, attitudes and practices regarding A(H1N1) pandemic influenza and its prevention by vaccination

Before the beginning of the campaign, 63% of paediatricians perceived A(H1N1) pandemic infection as a serious disease, that would occur frequently without vaccination comparatively to more than 75% after campaign initiation. In addition, less respondents considered that A(H1N1) pandemic influenza was severe enough to take special precautions to prevent it before, than after the start of the vaccination campaign (73% agreed or strongly agreed before compared to 63% after, p = 0.0136) (Table 2). Before the vaccination campaign, half of respondents or less agreed or strongly agreed that the A(H1N1) vaccine was safe (50%) and effective (35%) compared to 77% and 72% after the start of the campaign who felt the vaccine was safe and effective (p < 0.001) (Table 2). Paediatricians' perceived acceptability of A(H1N1) vaccine by the public remained comparable (45% before versus 41% after agreed and strongly agreed, p = 0.3608). Paediatricians' perceived acceptability of the A(H1N1) vaccine by health professionals who administered vaccines (hereafter named "vaccine providers") increased after the beginning of vaccination campaign (71% before versus 84% after, p < 0.001). Respondents' intention to recommend the A(H1N1) vaccine increased from 80% before the beginning of the campaign to 92% after, including 46% and 62% of paediatricians that declared they would strongly recommend it, respectively. Globally, 40% of respondents who disagreed with the usefulness to protect children with the seasonal influenza vaccine did not intended to recommend A(H1N1) pandemic vaccine to their patients while 2% of physicians who agreed with the usefulness of the seasonal influenza vaccine did not intended to recommend the A(H1N1) pandemic vaccine (p < 0.001) (data not shown). The majority of respondents intended to get vaccinated against A(H1N1) pandemic influenza themselves (84% before and 92% after, p = 0.003, Table 1). No statistically significant differences were observed in intention to be vaccinated between paediatricians practicing in different Canadian regions, neither before (p = 0.4315) nor after (p = 0.4291) the beginning of the campaign. Before the beginning of the vaccination campaign, 13% of paediatricians were undecided about being vaccinated themselves compared to 3% after. Finally, the proportion of paediatricians who reported they had received sufficient information on A(H1N1) vaccine increased from 31% before the beginning of the campaign to 73% after (p < 0.001), with 3% of respondents reporting they felt their knowledge was insufficient after the beginning of the vaccination campaign versus 17% before (Table 2).

**Table 2:** Paediatricians' knowledge, attitudes and practices regarding A(H1N1) pandemic influenza and vaccine (%)

	Before A(H1N1) vaccination campaign	After A(H1N1) vaccination campaign
**A(H1N1) pandemic influenza...**		
Is severe enough to take special precautions to prevent it	n = 704	n = 195
Somewhat agree	26.6	20.5
Agree	38.8	38
Strongly agree	24.3	34.9
Is a serious disease *	n = 671	n = 193
Somewhat agree	28,2	16.6
Agree	37.7	43
Strongly agree	25	36.3
Would occur frequently in Canada without vaccination *	n = 672	n = 190
Somewhat agree	25	14.2
Agree	36.9	36.8
Strongly agree	25.3	39
Generates a significant economic burden in Canada	n = 682	n = 190
Somewhat agree	15.3	9.5
Agree	33.3	34.2
Strongly agree	46.6	51.1
In my medical practice or in my private life, I have had experience with severe A(H1N1) pandemic influenza	n = 698	n = 193
Yes	59	81.3
**A(H1N1) pandemic influenza vaccines will be/are...**		
Safe *	n = 615	n = 186
Somewhat agree	42	20.4
Agree	34.3	45.7
Strongly agree	15.5	31.7
Effective *	n = 590	n = 187
Somewhat agree	54.6	25.1
Agree	27.1	43.9
Strongly agree	7.5	28.3
Well accepted by the public	n = 677	n = 187
Somewhat agree	35.9	41.2
Agree	29.4	34.2
Strongly agree	15.1	6.4
Well accepted by vaccine providers *	n = 671	n = 188
Somewhat agree	22.8	12.2
Agree	39.5	46.8
Strongly agree	31.7	37.2
I consider that my knowledge on the pandemic influenza (H1N1) vaccine is sufficient *	n = 690	n = 193
Somewhat agree	31	19.2
Agree	21	37.3
Strongly agree	10.4	35.2
I will recommend the A(H1N1) pandemic vaccine to my patients*	n = 671	n = 189
Somewhat agree	15.7	5.8
Agree	33.8	29.1
Strongly agree	46.1	62.4

* Before/after differences statistically significant at p < 0.05

### Factors associated with the intention to recommend A(H1N1) pandemic vaccine before and after the start of the vaccination campaign

The intention to get vaccinated against A(H1N1) pandemic influenza themselves (OR = 8.65) and belief that A(H1N1) pandemic vaccine would be well accepted by vaccine providers (OR = 6.65) were the most significant factors associated with the intention to recommend A(H1N1) pandemic vaccine to patients. Six other variables were also significantly associated with the intention to recommend the vaccine: belief that seasonal influenza vaccines are very useful to protect child health; perceived economic burden of A(H1N1) influenza illness; self-estimated sufficiency of knowledge about the A(H1N1) vaccine; perceived safety of the A(H1N1) vaccine; perceived severity of A(H1N1) pandemic influenza and belief that special precautions to prevent A(H1N1) pandemic influenza are needed (Table 3).

**Table 3:** Variables associated with respondents intention to recommend A(H1N1) pandemic vaccine in multivariate regression analysis (N = 709) *

Variables	Adjusted OR	95% CI	P value
Paediatricians' intention to receive A(H1N1) themselves	8.65	4.27-17.14	<0.0001
Belief^§ ^that A(H1N1) vaccine will be well accepted by vaccine providers	6.60	3.70-11.76	<0.0001
Belief^§ ^that seasonal influenza vaccines are very useful to protect children health	2.84	1.58-5.10	0.0005
Belief^§ ^that A(H1N1) pandemic influenza generate a significant economic burden in Canada	2.78	1.51-5.08	0.0010
Self-estimated sufficiency of knowledge on A(H1N1) vaccine	2.10	1.05-4.20	0.0351
Belief^§ ^that A(H1N1) vaccine will be safe	2.10	1.13-3.90	0.0195
Belief^§ ^that A(H1N1) pandemic influenza is a serious disease	2.09	1.13-3.88	0.0189
Belief^§ ^that A(H1N1) pandemic influenza is a severe enough to take special precautions to prevent it	1.88	1.04-3.42	0.0377
Subset variable (before = 1, after = 0)	0.84	0.36-1.96	0.6864

* Multivariate analyses; OR = odds ratio; CI = confidence interval; 205 physicians were excluded because of missing answers (170 before and 32 after)

^§ ^Strongly agree, agree

### Multiple correspondence analyses (MCA)

**Figure 1:**
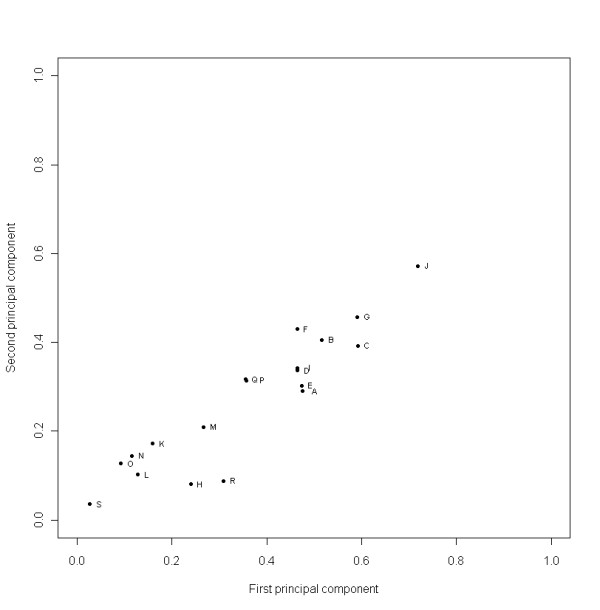
**Correlations' graph between variables and the first and second principal components**. Total percentage of the variability explained by first and second principal components was 13.6%. Variables almost uncorrelated with first and second principal components aren't represented on the graph, except subset variable (S). The variables: B, C, F, G, J contributed more to first and second principal components than others. - J: Respondent's intention to recommend A(H1N1) pandemic vaccine to their patients - G: "Belief that A(H1N1) vaccine will be effective" - F: "Belief that A(H1N1) vaccine will be safe" - C: "Belief that A(H1N1) pandemic influenza is a serious disease" - B: "Belief that seasonal influenza vaccines are very useful to protect children health" All variables, including variables almost uncorrelated with first and second principal component, are listed in an additional file (see Additional File 1).

Results of MCA supported the associations found in the logistic regression models (Figure 1). Overall, first and second principal components summarized 13.6% of the initial variables' variability. The coefficients of determination (r^2^) for the first and second principal components were low (maximum at 0.71 for the first principal components, and 0.57 for the second principal component). It showed that responses were homogeneous among respondents. More precisely, results showed that we could group our respondents by negative levels rather than positive levels of their answers: the levels "strongly disagree" and "disagree" were the most discriminatory levels.

Before and after the onset of the vaccination campaign, the modelled principal component had the respondents' intention to recommend A(H1N1) pandemic vaccine as principal contributor (weight for the "strongly disagree" level: 4.14). Then we found respondent's intention to receive A(H1N1) pandemic vaccine (weight for the 'no' level: 3.45), and "belief that seasonal influenza vaccines are very useful to protect children health" (weight for the "strongly disagree" level: 3.44). Mean of weight for all other variables is 0.87, including the subset variable which was the less correlated variable (variable S on figure 1) with first and second principal components.

## Discussion

To our knowledge, this is the first study that measured changes in physicians' KAP regarding A(H1N1) pandemic influenza and its prevention by vaccination before and after the approval of the vaccine and start of the vaccination campaign. Previous studies among health care workers assessed acceptability of the A(H1N1) vaccine before its official approval and program implementation or used hypothetical pandemic vaccination scenarios [18,22-26]. In these studies, intention to be vaccinated against A(H1N1) pandemic influenza varied from 48% to 80% among healthcare workers, comparatively to 84% of paediatricians surveyed before the beginning of the vaccination campaign in our study. Similarly to our results, a study done in Mexico reported that 72% of healthcare workers would recommend the vaccine to their patients, and they were more likely to do so when they had the intention to get vaccinated themselves [25].

Results of this national survey among paediatricians indicated an important increase in paediatricians perceptions of the burden of A(H1N1) pandemic influenza and support for A(H1N1) vaccination after the beginning of the vaccination campaign. Respondents' endorsement of almost all items regarding A(H1N1) pandemic influenza and its prevention by vaccination increased after the start of the vaccination campaign. This is not surprising given the fact that the first A(H1N1) vaccine available in Canada used a novel adjuvant (AS03) for which limited information regarding the safety and immunogenicity was available. The proportion of physicians who reported they had received sufficient information about A(H1N1) vaccine also increased by 42% after the start of the vaccination campaign. This increase may be attributable to the important educational efforts done at the beginning of the vaccination campaign along with the official recommendations by expert groups and professional associations that were released in early November [27-29].

Health professionals' knowledge about vaccines has been previously shown as a main determinant associated with their own vaccine uptake and their intention to recommend the vaccine to their patients [30,31]. The association between physicians own vaccination behaviours and their recommendations to their patients was previously established [12,32-35]. It appears to hold true in a pandemic context, as shown by our results and those previously reported in Mexico [25].

Our results highlight the positive change in paediatricians' knowledge and level of support of the A(H1N1) vaccines throughout the pandemic vaccination campaign. This change may be attributable to increased education efforts and the very rare vaccine associated adverse events [36], but may also reflect the intense media attention focused on the vaccination campaign. A recent UK study has shown that healthcare workers were more willing to accept stockpiled H5N1 vaccine during a period of high media coverage of a H5N1 outbreak in a poultry farm than 6 months after (63.4% vs. 51.9%, p = 0.009) [18]. The increased exposure of paediatricians to severe cases of A(H1N1) disease, as observed in our results, may also have enhanced paediatricians' acceptability of A(H1N1) pandemic vaccine.

In logistic regression analysis, paediatricians' intention to get vaccinated against A(H1N1) pandemic influenza themselves was the most significant factor associated with the intention to recommend A(H1N1) pandemic vaccine to patients. Results obtained by the multiple correspondence analysis (MCA) are consistent with results from the logistic regression analysis. The negative levels were also more discriminatory than the positive ones. This is consistent with results of previous studies that have shown that knowledge and behaviors regarding seasonal influenza influenced A(H1N1) vaccination status: individuals who were not vaccinated against seasonal influenza were less likely to have the intention to receive the pandemic vaccine [37,38]. Intention to be vaccinated against A(H1N1) pandemic influenza was also higher than vaccine uptake against seasonal influenza among healthcare workers usually reported in Canadian studies [39,40], which was estimated at 64% in 2006 [41]. In our study, a significant proportion of paediatricians who disagreed with the usefulness to protect children with the seasonal influenza vaccine did not intended to recommend the A(H1N1) vaccine to their patients. However, results obtained by MCA should be interpreted with precautions as only 13.6% of the variability is summarized by the first and second principal components. This is principally due to the uneven distribution of response levels.

Our study has several limitations. First, the study wasn't initially designed for a "before-after" analysis. The increase in the willingness to be vaccinated against A(H1N1) pandemic influenza observed among paediatricians may result from a response bias of respondents having more doubts about pandemic vaccination before the vaccination campaign actually started. Nonetheless, respondents' demographic and professionals characteristics as well as their attitudes toward vaccination in general were very similar, thus suggesting the two subsets of participants were comparable. Second, the dichotomization of the dependant variable ("strongly agree" and "agree" versus all others) was a conservative choice and physicians who answered "somewhat agree" were considered as having a neutral opinion, not a positive one. Third, the repartition of the answers to the dependant variable in the subset of data collected after the beginning of the vaccination campaign did not allow us to perform two multivariate analysis for the two subset. However, the model was adjusted to take into consideration the time period when the paediatricians completed the survey (before or after the initiation of the campaign). Finally, the response rate was 50% and a non-participation bias cannot be excluded. However, the response rate remains satisfactory for a mail-based survey with physicians [42-44]. In addition, socio-demographic characteristics of respondents are comparable to those reported in other surveys conducted among Canadian paediatricians [42,45]. Socio-professional characteristics of respondents also allow us to suppose a good representativeness.

Results of this study indicated a high level of paediatricians willingness to be vaccinated against A(H1N1) and to recommend the vaccine to their patients. Lack of knowledge on A(H1N1) vaccine, belief that A(H1N1) was not a severe disease as well as concerns over A(H1N1) vaccine safety and usefulness were barriers to paediatricians' intention to recommend it. This is consistent with barriers to seasonal influenza vaccination among healthcare workers reported in the literature [31,46,47]. Public health interventions to promote seasonal influenza vaccination among healthcare workers should include the delivery of evidence-based information regarding influenza vaccines' safety, efficacy and usefulness. Educational campaigns should also stress out the threat posed by seasonal influenza to healthcare workers and the patients.

## Conclusion

In summary, the results show the important increases in physicians' level of confidence about A(H1N1) vaccine safety and immunogenicity and their willingness to recommend this vaccine during the first months of the campaign. More than 40% of all Canadians aged 12 years or older received at least one dose of the A(H1N1) vaccine during the vaccination campaign [48]. In the province of Quebec, Canada, almost 80% of the children aged between 6 months and 5 years were vaccinated against A(H1N1) influenza [49]. Paediatricians' support of the vaccination campaign and their recommendations were surely one of the key components of such a success.

## Competing interests

This study was financially supported by the Quebec Ministry of Health and Social Services and by an unrestricted grant from GlaxoSmithKline. No private company or their employees were involved in study protocol/questionnaire designing, data collection, data analysis and interpretation or manuscript writing.

## Authors' contributions

All authors except FD have been involved in the design of the study. ED and FD have drafted the manuscript. FD performed the statistical analysis. All authors have read and approved the final version of the manuscript.

## Pre-publication history

The pre-publication history for this paper can be accessed here:

http://www.biomedcentral.com/1471-2458/11/128/prepub
